# Dehydroepiandrosterone sulfate and dehydroepiandrosterone sulfate/cortisol ratio in cirrhotic patients with septic shock: another sign of hepatoadrenal syndrome?

**DOI:** 10.1186/s13054-017-1768-0

**Published:** 2017-08-15

**Authors:** Ming-Hung Tsai, Hui-Chun Huang, Yun-Shing Peng, Yung-Chang Chen, Ya-Chung Tian, Chih-Wei Yang, Jau-Min Lien, Ji-Tseng Fang, Cheng-Shyong Wu, Sen-Yung Hsieh, Fa-Yauh Lee

**Affiliations:** 1 Division of Gastroenterology and Hepatology, Chang Gung Memorial Hospital, Chang Gung University, Taoyuan, Taiwan; 2 Division of Gastroenterology and Hepatology, Department of Internal Medicine, Taipei Veteran General Hospital, No. 201, Section 2, Shih-Pai Road, Taipei, 11217 Taiwan; 3 Faculty of Medicine, Yang-Ming University School of Medicine, Taipei, Taiwan; 4 Division of General Medicine, Department of Medicine, Taipei Veteran General Hospital, Taipei, Taiwan; 5 Division of Endocrinology, Chang Gung Memorial Hospital, Chia-Yi, Taiwan; 6 Chang Gung University, Taoyuan, Taiwan; 7 Division of Critical Care Nephrology, Kidney Institute, Chang Gung Memorial Hospital, Taipei, Taiwan; 8 Division of Gastroenterology, Chang Gung Memorial Hospital, Chia-Yi, Taiwan

**Keywords:** Adrenal androgen, Steroidogenesis, Cirrhosis, Sepsis

## Abstract

**Background:**

Cirrhotic patients are susceptible to sepsis and critical illness-related corticosteroid insufficiency (CIRCI). Dehydroepiandrosterone sulfate (DHEAS) is a corticotropin-dependent adrenal androgen, which has immunostimulating and antiglucocorticoid effects. Considering the synchronized synthesis of cortisol and DHEAS and their opposing effects to each other, investigators have proposed measuring these two hormones as a ratio. Severe sepsis has been associated with low DHEAS, especially relative to high cortisol. Despite growing interest in the role of adrenal androgen replacement in critical illness, there have been no data about DHEAS and the DHEAS/cortisol ratio in patients with liver cirrhosis. We studied whether low concentrations of DHEAS and decreased DHEAS/cortisol ratio are associated with poor outcome in patients with liver cirrhosis and septic shock.

**Methods:**

We recruited 46 cirrhotic patients with septic shock, and 46 noncirrhotic counterparts matched by age and sex. We evaluated adrenal function using the short corticotropin stimulation test and analyzed the relation between DHEAS and cortisol.

**Results:**

While the nonsurvivors in the cirrhotic group had significantly lower baseline DHEAS, lower baseline DHEAS/cortisol ratio, and reduced increments of both DHEAS and cortisol upon corticotropin stimulation, the survivors had lower baseline cortisol. Cirrhotic patients with lower DHEAS/cortisol ratio (<1.50) had higher levels of interleukin-6 and tumor necrosis factor alpha, higher Sequential Organ Failure Assessment scores, and higher rates of CIRCI and hospital mortality. Using the area under the receiver operating characteristic (AUROC) curve, both DHEAS and the DHEAS/cortisol ratio demonstrated a good discriminative power for predicting hospital survival (AUROC 0.807 and 0.925 respectively). The cirrhotic group had lower DHEAS and DHEAS/cortisol ratio but higher rates of CIRCI and hospital mortality, compared to the noncirrhotic group.

**Conclusions:**

There is dissociation between cortisol (increased) and DHEAS (decreased) in those cirrhotic patients who succumb to septic shock. Low DHEAS/cortisol ratios are associated with more severe diseases, inflammation, and CIRCI and can serve as a prognostic marker. More investigations are needed to evaluate the role of adrenal androgen in this clinical setting.

## Background

Septic shock is accompanied by activation of the hypothalamic–pituitary–adrenal (HPA) axis, which is highlighted by increased serum corticotropin and glucocorticoid [1–3]. Parallel to glucocorticoid secretion, HPA activation leads to the release of dehydroepiandrosterone (DHEA) and its sulfate (DHEAS). Both of these are adrenal androgens and practically all is secreted by the adrenal glands [4]. The serum concentration of DHEAS is 300–500 times higher than that of DHEA and can be considered a circulating reservoir [4]. In contrast to serum cortisol or DHEA concentrations, serum DHEAS levels do not exhibit a diurnal variation, as a consequence of a longer half-life [4]. Therefore, serum DHEAS levels have been proposed to serve as a potential biomarker of adrenal function in different clinical settings [5, 6]. In fact, investigators have combined adrenocorticotropic hormone (ACTH) stimulation tests or insulin tolerance tests with measurements of DHEAS to define normality of the HPA axis [7, 8]. Importantly, similar to that of glucocorticoid, the secretion of adrenal androgen is almost exclusively under the trophic effect of ACTH [4]. Normally, adrenal androgen is secreted synchronously with glucocorticoid from the adrenal cortex [9]. Although the specific physiological function of DHEA is still unclear, it has been shown that DHEA modulates the function of the immune system. DHEA plays an important role in the interaction between the endocrine and immune systems [10, 11]. The immune-modulatory properties of DHEA include induction of T-cell activation and interleukin-2 production [12, 13], and enhancing cytotoxic functions of monocytes [14]. Because of these immune-enhancing effects, adrenal androgen has been considered a functional antagonist of glucocorticoids, which can be immunosuppressive [4]. In fact, a recent study indicated that DHEA and glucocorticoids regulate the same immune-modulating genes in opposite directions [15]. Considering lines of evidence for activities of adrenal androgen that offset those of glucocorticoid, investigators have proposed quantification of their levels as a ratio, to serve as an alternative index of adrenal activity in different clinical settings [16, 17]. Interestingly, the responses of glucocorticoid and androgen demonstrate a significant discrepancy during critical illness, with adrenal androgen levels decreasing and glucocorticoid levels increasing [18]. This phenomenon is more pronounced in most severely ill patients and nonsurvivors [18–20], suggesting that functional adaptation of adrenal steroidogenesis may exhaust the counterregulatory mechanisms between adrenal androgen and glucocorticoid, thus negatively impacting the prognosis of critical illness. Indeed, an increased cortisol to DHEAS ratio was shown in nonsurvivors [19, 20], indicating that an exhausted adrenal reserve can serve as a prognostic factor.

Liver cirrhosis is significantly associated with an increased risk of sepsis and sepsis-related mortality [21, 22]. Indeed, upregulated inflammatory cytokines, which mediate inflammation and organ dysfunction in the setting of liver cirrhosis and sepsis [22, 23], are major components facilitating interaction between immune and neuroendocrine systems [24]. Critical illness-related corticosteroid insufficiency (CIRCI) or relative adrenal insufficiency is common in cirrhotic patients with severe sepsis and septic shock, and is associated with increased mortality [25–28]. However, relevant data about adrenal androgen in critically ill cirrhotic patients are still lacking despite growing interest in the role of adrenal androgen replacement in critical illness [29]. In fact, decreased levels of adrenal androgen are reported in noncritical patients with liver cirrhosis [30, 31]. In a sense, overshooting inflammation in septic shock may overwhelm the adaptive strategy of adrenal steroidogenesis in those patients who succumb. We hypothesized that defective DHEAS production and adrenal exhaustion are associated with inflammation and poor prognosis in cirrhotic patients with septic shock. In this prospective observational investigation, we used the short corticotropin stimulation test (SST) to evaluate adrenal function and studied whether low concentrations of DHEAS and a decreased DHEAS/cortisol ratio are associated with poor outcome in patients with liver cirrhosis and septic shock.

## Methods

### Patient information, data collection, and definitions

This study was conducted with the approval of the institutional review board of Chang Gung Memorial Hospital, Taiwan and in accordance with the Declaration of Helsinki of the World Medical Association. Written informed consent was obtained from the patients or from their legally accepted representatives for those with hepatic encephalopathy. From August 2010 to January 2012, 46 cirrhotic patients were consecutively enrolled and fulfilled the criteria of septic shock proposed by the members of American College of Chest Physicians/Society of Critical Care Medicine consensus conference committee [32], namely sepsis-induced hypotension despite adequate fluid resuscitation, along with the presence of hypoperfusion abnormalities associated with organ dysfunction. Sepsis-induced hypotension was defined as systolic blood pressure < 90 mmHg or a reduction < 40 mmHg from baseline in the absence of other causes for hypotension. Liver cirrhosis was defined histologically or based on clinical, image, and laboratory findings. The diagnosis was made histologically in eight patients in whom liver biopsy was performed to diagnose hepatocellular carcinoma or confirm liver cirrhosis. Liver cirrhosis was diagnosed clinically in 38 patients. A control group of 46 patients with septic shock without cirrhosis, matched by age and sex, was identified from patients admitted during the same period. All patients were resuscitated with a standard treatment protocol for management of septic shock [33].

The severity of liver disease on the day of the SST was graded by the Child–Pugh system and the Model for End-stage Liver Disease (MELD) [34, 35]. Meanwhile, illness severity was also assessed by Sequential Organ Failure Assessment (SOFA) [36]. For these scoring systems and physiological evaluations, the most abnormal value for each organ system on the day of the SST was recorded.

Patients with a history of corticosteroid treatment and those who had received the steroidogenesis-inhibiting agent etomidate over the preceding 6 months were excluded from this study. The main outcome analyzed was hospital mortality.

### Laboratory investigations

Hematological and biochemical data were collected systemically on the day of the SST. Blood cultures, urine sediment, urine culture, ascitic fluid neutrophil count, and culture were performed routinely at inclusion. Blood cultures and appropriate culture specimens from the infection focus were obtained during hospitalization if necessary.

A SST was performed within the first 24 hours of admission to the ICU. Synthetic ACTH (250 μg, Synacthen; Novartis Pharma AG, Basel, Switzerland) was given intravenously. Blood samples were obtained immediately before and 30 and 60 minutes after injection. Cortisol levels were measured by a competitive immunoassay using direct chemiluminescent technology (Roche Diagnostics, Mannhein, Germany). DHEAS levels were measured using a highly specific immunoradiometric assay (Nichols Institute Diagnostics, San Clemente, CA, USA). For cortisol measurement, the intra-assay coefficient of variation (CV) was 2.8% and the inter-assay CV was 3.6%. For DHEAS, the intra-assay CV was 8.6% and the inter-assay CV was 9.8%. The peak levels of cortisol or DHEAS were defined as the highest levels obtained following synacthen administration, whether at 30 or 60 minutes. The cortisol or DHEAS increment was defined as the difference between the baseline and peak cortisol or DHEAS level. According to the consensus statements from an international task force [37], the criteria for CIRCI are defined as follows: baseline value < 10 μg/dl or cortisol response < 9 μg/dl. The concentrations of TNF-α and IL-6 were measured by an enzyme-linked immunosorbent assay (R & D Systems, Minneapolis, MN, USA).

### Statistical analysis

Descriptive statistics are expressed as mean ± SD or median (interquartile range (IQR)). All variables were tested for normal distribution using the Kolmogorov–Smirnov test. The Student *t* test was used to compare the means of continuous variables and the normal distribution data. Otherwise, the Mann–Whitney *U* test was used. Categorical data were tested using the chi-square (χ^2^) test. The correlation between the results of the SST and the disease severity scores was analyzed with linear regression using the Pearson method. Discrimination was tested using the area under a receiver operating characteristic (ROC) curve to assess the ability of DHEAS and the DHEAS/cortisol ratio to predict hospital survival. ROC analysis was also performed to calculate the cutoff values, sensitivity, specificity, overall correctness, and positive and negative predictive values. The best Youden index (sensitivity + specificity – 1) was also used to determine the best cutoff point of DHEAS and DHEAS/cortisol ratio to predict hospital survival. All statistical tests were two-tailed, and the significance level was set at *p* = 0.05 or lower. Data were analyzed using SPSS 13.0 for Windows (SPSS Inc., Chicago, IL, USA) except for comparisons of ROC curves. The comparisons between ROC curves were calculated with MedCalc software (MedCalc Software 14.12.0, Belgium), using Hanley and McNeil’s method [38].

## Results

### Subjects’ characteristics and short corticotropin stimulation test

During the study period, 46 cirrhotic patients with septic shock were consecutively recruited and evaluated. Overall, the hospital mortality for the entire group was 73.9%.

**Fig. 1:**
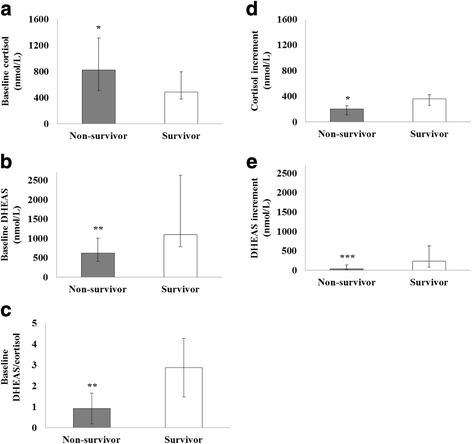
Results of SSTs. **a** Levels of baseline cortisol are significantly higher in nonsurvivors. **b** Levels of baseline DHEAS are significantly higher in survivors. **c** Baseline DHEAS/cortisol ratios are significantly higher in survivors. **d** Cortisol increments upon challenge of ACTH are significantly higher in survivors. **e** DHEAS increments upon challenge of ACTH are significantly higher in survivors. Results expressed as median, *error bars* representing the interquartile range, in **a**, **b**, **d**, **e**. Results expressed as mean, *error bars* representing the standard deviation, in **c**. *p < 0.05, **p < 0.01, ***p < 0.001. *DHEAS* dehydroepiandrosterone sulfate

Table 1 presents patient demographic data, clinical characteristics, and results of the SST for both survivors and nonsurvivors. As shown in Table 1 and Fig. 1, both cortisol and DHEAS increments upon challenge of ACTH were significantly higher in those who survived. While the baseline cortisol levels were higher in those who died, the baseline DHEAS levels were significantly higher in those who survived. As a consequence, the baseline DHEAS/cortisol ratio was significantly lower in those who died. The peak cortisol levels were not different between survivors and nonsurvivors, while peak DHEAS levels were significantly lower in nonsurvivors. Both IL-6 and TNF-α levels were significantly higher in nonsurvivors. The discriminating power of baseline DHEAS and DHEAS/cortisol ratio to predict hospital survival was tested using the area under a ROC curve. The areas under ROC curves (mean ± SEM) for baseline DHEAS and DHEAS/cortisol ratio were 0.807 ± 0.071 (95% CI: 0.668–0.947) and 0.925 ± 0.039 (95% CI: 0.848–1.002) respectively. Comparison of AUROCs using Hanley and McNeil’s method showed that the baseline DHEAS/cortisol ratio gave a significantly higher AUROC (*p* = 0.032) and thus better predictive accuracy than baseline DHEAS. Table 2 presents the predictive values of the chosen cutoff points (755 nmol/L for DHEAS, 1.50 for DHEAS/cortisol ratio), which give the best Youden index, for prediction of hospital survival. The clinical characteristics and outcomes in patient subgroups stratified by DHEAS/cortisol ratio are presented in Table 3. As this table shows, a low DHEAS/cortisol ratio is associated with higher SOFA scores, inflammatory cytokine levels, rates of CIRCI, and hospital mortality.

**Table 1:** Patients’ demographic data and clinical characteristics at admission to the ICU grouped according to hospital mortality

	Hospital survivors (*n* = 12)	Hospital nonsurvivors (*n* = 34)	*p* value
Age (years)	47.6 ± 14.8	58.8 ± 14.3	0.029
Gender (M/F)	11/1	25/9	0.252
SOFA score	8.3 ± 2.5	14.0 ± 3.6	<0.001
MELD score	20.9 ± 10.7	33.0 ± 9.9	0.001
Child–Pugh score	10 (7.3–11.8)	12 (11–13.3)	0.005
ACTH (pg/ml)	8.7 (7.5–25.5)	20.7 (13.1–35.1)	0.084
Baseline cortisol (nmol/L)	484 (381–796)	821 (508–1312)	0.026
Peak cortisol (nmol/L)	955 (633–1258)	993 (807–1549)	0.354
Cortisol increment (nmol/L)	359 (257–428)	200 (110–252)	0.011
Baseline DHEAS (nmol/L)	1099 (787–2627)	622 (403–1006)	0.002
Peak DHEAS (nmol/L)	1286 (1118–2874)	699 (429–1115)	<0.001
DHEAS increment (nmol/L)	236 (179–391)	41 (10–96)	<0.001
Baseline DHEAS/cortisol ratio	2.86 ± 1.40	0.92 ± 0.73	0.001
IL-6 (pg/ml)	110 (54–233)	571 (247.25–924)	0.004
TNF-α (pg/ml)	10 (5.5–25)	45.5 (31.25–78.25)	0.001

Data presented as mean ± SD, number, or median (interquartile range)

*M* male, *F* female, *SOFA* Sequential Organ Failure Assessment, *MELD* Model for End-Stage Liver Disease, *ACTH* adrenocorticotropic hormone, *DHEAS* dehydroepiandrosterone sulfate, *IL-6* interleukin-6, *TNF-*α tumor necrosis factor alpha, *ICU* intensive care unit

**Table 2:** Baseline DHEAS and DHEAS/cortisol ratio to predict hospital survival

	Sensitivity	Specificity	PPV	NPV	Accuracy
DHEAS	0.818	0.647	0.429	0.917	0.689
DHEAS/cortisol	0.909	0.842	0.625	0.966	0.844

*DHEAS* dehydroepiandrosterone sulfate, *PPV* positive predictive value, *NPV* negative predictive value

**Table 3:** Patients’ demographic data and clinical characteristics grouped according to baseline DHEAS/cortisol ratio

	High DHEAS/cortisol ratio (>1.50) (*n* = 17)	Low DHEAS/cortisol ratio (<1.50) (*n* = 29)	*p* value
Age (years)	48.7 ± 11.4	60.2 ± 15.2	0.010
Gender (M/F)	15/2	21/8	0.282
Hospital mortality	6/17 (35.3%)	28/29 (96.6%)	<0.001
SOFA score	10.8 ± 4.5	13.6 ± 3.7	0.030
MELD score	26.1 ± 12.7	32.1 ± 10.0	0.082
Child–Pugh score	12 (8–12.5)	12 (11–13.5)	0.106
ACTH (pg/ml)	19 (7.8–27.2)	20.4 (10.8–38)	0.372
Baseline cortisol (nmol/L)	441 (382–691)	910 (661–1422)	0.001
Peak cortisol (nmol/L)	822 (608–1141)	1048 (902–1671)	0.006
Cortisol increment (nmol/L)	273 (189–403)	196 (97–288)	0.037
Baseline DHEAS (nmol/L)	1099 (885–2124)	551 (362–769)	<0.001
Peak DHEAS (nmol/L)	1286 (974–2546)	643 (360–1057)	<0.001
DHEAS increment (nmol/L)	135.7 (88.2–248.3)	35.3 (6.8–118.1)	0.009
IL-6 (pg/ml)	198 (58.5–444)	571 (221–1012)	0.025
TNF-α (pg/ml)	17.6 (8.4–32.3)	47.5 (30–96.8)	0.002
CIRCI	7/17 (41.2%)	21/29 (72.4%)	0.036

Data presented as mean ± SD, number (percentage), or median (interquartile range)

*M* male, *F* female, *SOFA* Sequential Organ Failure Assessment, *MELD* Model for End-Stage Liver Disease, *ACTH* adrenocorticotropic hormone, *DHEAS* dehydroepiandrosterone sulfate, *IL-6* interleukin-6, *TNF-*α tumor necrosis factor alpha, *CIRCI* critical illness-related corticosteroid deficiency

According to the criteria stated, 28 (60.8%) patients had CIRCI. The clinical characteristics and outcomes in patient subgroups stratified by adrenal functions are presented in Table 4. Those patients with CIRCI had lower baseline DHEAS levels and DHEAS/cortisol ratios and higher levels of inflammatory cytokines.

**Table 4:** Patients’ demographic data, clinical characteristics at admission to ICU and outcomes grouped according to the SST

	CIRCI (*n* = 28)	Non-CIRCI (*n* = 18)	*p* value
Age (years)	56.0 ± 12.8	56.0 ± 18.0	0.997
Gender (M/F)	22/6	14/4	1.000
SOFA score	14.8 ± 3.3	8.7 ± 2.8	<0.001
MELD score	35.3 ± 8.8	21.3 ± 9.5	<0.001
Child–Pugh score	13 (12–14)	9 (8–11.5)	<0.001
ACTH (pg/ml)	28 (15–40)	7.9 (6.7–18.3)	<0.001
Baseline cortisol (nmol/L)	877 (481–1331)	555 (417–859)	0.054
Peak cortisol (nmol/L)	967 (695–1505)	1021 (803–1458)	0.727
Cortisol increment (nmol/L)	145 (70–217)	399 (314–546)	<0.001
Baseline DHEAS (nmol/L)	678 (437–1111)	921 (524–1552)	0.229
Peak DHEAS (nmol/L)	750 (491–1141)	1159 (595–1866)	0.075
DHEAS increment (nmol/L)	41 (16–101)	180 (90–278)	0.002
Baseline DHEAS/cortisol	1.01 ± 0.79	2.11 ± 1.58	0.012
IL-6 (pg/ml)	660 (240–968)	164 (85–328)	0.011
TNF-α (pg/ml)	44 (31–79)	16 (6–36)	0.007
Hospital mortality	26/28 (92.9%)	8/18 (44.4%)	<0.001

Data presented as mean ± SD, number (percentage), or median (interquartile range)

*M* male, *F* female, *SST* short corticotropin stimulation test, *CIRCI* critical illness-related corticosteroid deficiency, *SOFA* Sequential Organ Failure Assessment, *MELD* Model for End-Stage Liver Disease, *ACTH* adrenocorticotropic hormone, *DHEAS* dehydroepiandrosterone sulfate, *IL-6* interleukin-6, *TNF-*α tumor necrosis factor alpha, *ICU* intensive care unit

**Fig. 2:**
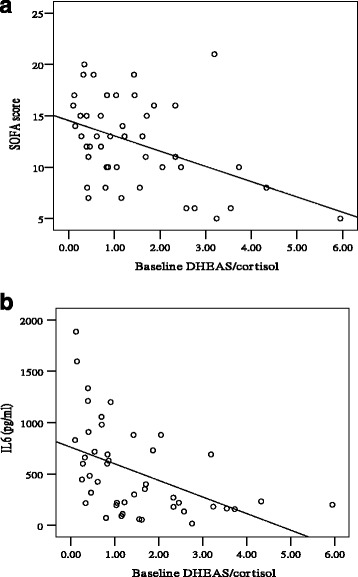
**a** Baseline DHEAS/cortisol ratio is negatively correlated to SOFA score (*R* = –0.440, *p* = 0.002). **b** Baseline DHEAS/cortisol ratio is negatively correlated to IL-6 (*R* = –0.503, *p* = 0.005). *SOFA* Sequential Organ Failure Assessment, *DHEAS* dehydroepiandrosterone sulfate, *IL6* interleukin-6

Cortisol and DHEAS increments were positively correlated (*R* = 0.378, *p* = 0.010). Both cortisol and DHEAS increments were inversely correlated to SOFA score (*R* = –0.517, *p* < 0.001 and *R* = –0.476, *p* = 0.001 respectively), MELD score (*R* = –0.444, *p* = 0.002 and *R* = –0.382, *p* = 0.009 respectively), and Child–Pugh score (*R* = –0.546, *p* < 0.001 and *R* = –0.368, *p* = 0.012 respectively). While baseline cortisol levels were inversely correlated to cortisol increments (*R* = –0.382, *p* = 0.009), baseline DHEAS levels were positively correlated to DHEAS increments (*R* = 0.521, *p* < 0.001). The baseline DHEAS/cortisol ratio was negatively correlated to SOFA score (*R* = –0.440, *p* = 0.002) (Fig. 2a), MELD score (*R* = –0.371, *p* = 0.011), Child–Pugh score (*R* = –0.471, *p* = 0.001), IL-6 (*R* = –0.503, *p* = 0.005) (Fig. 2b), and TNF-α (*R* = –0.634, *p* < 0.001).

### Comparison between patients with septic shock with and without liver cirrhosis

Table 5 presents the comparison between two groups of patients with and without cirrhosis matched by age and sex. Patients with liver cirrhosis and septic shock had significantly lower levels of baseline DHEAS and DHEAS/cortisol ratios and significantly higher rates of hospital mortality. While the cirrhotic group had significantly lower increments of cortisol and DHEAS, the noncirrhotic group had lower levels of TNF-α and IL-6.

**Table 5:** Comparison between cirrhotic and noncirrhotic groups matched according to age and sex

	Liver cirrhosis (*n* = 46)	Nonliver cirrhosis (*n* = 46)	*p* value
Age (years)	56.0 ± 14.9	58.1 ± 20.5	0.556
Gender (M/F)	36/10	36/10	1.000
SOFA score	12.7 ± 4.1	6.7 ± 4.1	<0.001
Baseline cortisol (nmol/L)	748 (450–1203)	778 (590–1109)	0.628
Peak cortisol (nmol/L)	993 (744–1479)	1195 (948–1589)	0.041
Cortisol increment (nmol/L)	221 (121–348)	408 (285–570)	<0.001
ACTH (pg/ml)	19.1 (9.1–34.0)	20.3 (8–30.5)	0.842
Baseline DHEAS (nmol/L)	703 (464–1180)	842 (587–1432)	<0.001
Peak DHEAS (nmol/L)	939 (497–1296)	1037 (643–1752)	<0.001
DHEAS increment (nmol/L)	89.6 (23–185)	325.7 (174–546)	<0.001
Baseline DHEAS/cortisol	1.40 ± 1.24	3.86 ± 2.57	0.001
IL-6 (pg/ml)	424 (206–855)	88 (35–275)	0.002
TNF-α (pg/ml)	34 (21–70)	15 (10.65–23.5)	0.003
Hospital mortality	34/46 (73.9%)	16/46 (34.8%)	<0.001

Data presented as mean ± SD, number (percentage), or median (interquartile range)

*M* male, *F* female, *SOFA* Sequential Organ Failure Assessment, *ACTH* adrenocorticotropic hormone, *DHEAS* dehydroepiandrosterone sulfate, *IL-6* interleukin-6, *TNF-*α tumor necrosis factor alpha

### Comparison between male and female patients with liver cirrhosis

To address the gender effects on DHEAS biosynthesis, we stratified cirrhotic patients into male and female groups. As shown in Table 6, there was no difference in levels of baseline DHEAS, peak DHEAS, DHEAS increment, and baseline DHEAS/cortisol between male and female groups.

**Table 6:** Patients’ demographic data and clinical characteristics at admission to ICU grouped according to gender

	Male (*n* = 36)	Female (*n* = 10)	*p* value
Age (years)	52.9 ± 12.6	67.0 ± 17.9	0.007
Hospital mortality	25/36 (69.4%)	9/10 (90%)	(0.252)
SOFA score	12.2 ± 4.4	13.0 ± 4.2	(0.618)
MELD score	30.5 (24–39.8)	27 (16.8–37.8)	(0.431)
Child–Pugh score	12 (10.3–13)	11.5 (9–13.3)	(0.666)
ACTH (pg/ml)	19.1 (9.8–32.7)	22 (6.3–47.9)	(0.929)
Baseline cortisol (nmol/L)	675 (428–1086)	975 (680–1260)	(0.132)
Peak cortisol (nmol/L)	927 (697–1381)	1338 (955–1639)	(0.088)
Cortisol increment (nmol/L)	221 (112–353)	240 (167–466)	(0.432)
Baseline DHEAS (nmol/L)	704 (437–1140)	824 (510–1428)	(0.409)
Peak DHEAS (nmol/L)	877 (491–1263)	961 (510–1692)	(0.489)
DHEAS increment (nmol/L)	89.6 (25.8–171.7)	70.6 (0–245.7)	(0.957)
Baseline DHEAS/cortisol ratio	1.46 ± 1.16	1.37 ± 1.68	(0.843)
IL-6 (pg/ml)	318 (150–855)	482 (310–1288)	(0.404)
TNF-α (pg/ml)	31 (11.2–78)	47 (39–52.5)	(0.208)
CIRCI	22/36 (61.1%)	6/10 (60%)	(1.000)

Data presented as mean ± SD, number (percentage), or median (interquartile range)

*SOFA* Sequential Organ Failure Assessment, *MELD* Model for End-Stage Liver Disease, *ACTH* adrenocorticotropic hormone, *DHEAS* dehydroepiandrosterone sulfate, *IL-6* interleukin-6, *TNF-*α tumor necrosis factor alpha, *CIRCI* critical illness-related corticosteroid deficiency, *ICU* intensive care unit

## Discussion

The major findings of this study are as follows. First, the ratio of DHEAS (an immunostimulant) to cortisol (an immunosuppressant) is significantly lower in patients with liver cirrhosis and septic shock, compared to their noncirrhotic counterparts. Second, upon admission to the ICU there is a dissociation between baseline DHEAS (reduced) and cortisol (increased) in nonsurvivors of the cirrhotic group, suggesting a functional adaptation of adrenal steroidogenesis in this subgroup. Finally, upon admission to the ICU a low DHEAS/cortisol ratio is associated with CIRCI and hospital mortality. The DHEAS/cortisol ratio can serve as a prognostic marker in this clinical setting.

Considering the synchronized synthesis of cortisol and DHEAS and their opposing effects to each other, there has been growing interest in measuring these two hormones as a ratio rather than as separate values [16, 17]. In this regard, our study is the first to measure the ratio of DHEAS to cortisol in liver cirrhosis. Interestingly, we found that a low DHEAS/cortisol ratio is associated with inflammation, disease severity, CIRCI, and hospital mortality (Table 3). Our results suggest that the balance between these two mutually dependent hormones may influence the pathophysiological process and ultimately outcomes. Indeed, this index has been used as an indicator in different diseases [16, 17, 19, 20]. This approach also helps to conceptualize the preferential synthesis of glucocorticoid over adrenal androgen in liver cirrhosis and septic shock. Previous studies have shown that there is a discrepancy between DHEAS (decreased) and cortisol (increased) in critical illness [18]. In accordance with this notion, we showed that nonsurvivors of the cirrhotic group had significantly lower baseline DHEAS and significantly higher baseline cortisol, and thus a lower DHEAS/cortisol ratio. The combination of low DHEAS levels with high cortisol levels suggested a shift in pregnenolone metabolism away from adrenal androgen pathways toward the glucocorticoid pathway in this subset of patients. It is conceivable that this functional adaptation could serve to minimize the synthesis of adrenal androgen and maximize the secretion of glucocorticoid which is acutely necessary to antagonize inflammation. Other intriguing findings are that baseline cortisol levels were negatively correlated to cortisol increments, whereas baseline DHEAS levels were positively correlated to DHEAS increments. Furthermore, both DHEAS and cortisol increment were negatively correlated to disease severity scores and associated with hospital mortality. Taken together, preferential adaptation of adrenal steroidogenesis favoring glucocorticoid pathway may deplete adrenal reserve and exhaust the counterbalance between adrenal androgen and glucocorticoid, thus negatively impacting the prognosis of liver cirrhosis with septic shock.

Adrenal androgens are pleiotropic hormones with multiple biological functions. In addition to the immune-enhancing effects mentioned previously, adrenal androgen has been shown to modulate cardiovascular functions. DHEA can exert its protective effects on the cardiac function and integrity of microcirculation of different vasculatures through activating Akt–eNOS signaling pathways [39–42]. Indeed, in experimental animals subjected to sepsis, administration of DHEA can attenuate organ dysfunction and improve survival. This effect was paralleled by decreased inflammatory cytokine levels and an improved activity of T-cell-mediated immunity [10, 43]. Moreover, salutary effects on hepatic perfusion were also demonstrated in experimental animals treated with androstenediol, a metabolite of DHEA, and subjected to trauma-hemorrhagic shock [44]. Interestingly, these beneficial effects of androstenediol on intrahepatic hemodynamics are due to induction of eNOS-mediated NO and a decrease in endothelin-1 [44], which may also represent a potential remedy for endothelial dysfunction in the microcirculation of cirrhotic liver [45]. It is unknown whether adrenal androgen can provide beneficial effects on immunity and microcirculation and thus improve outcomes in liver cirrhosis with septic shock.

Recently, CIRCI or relative adrenal insufficiency has been used to describe a suboptimal adrenal response to ACTH in critical illness, in which the glucocorticoid levels, although high in terms of absolute value, are inadequate to control inflammation [1, 37]. In most studies, only glucocorticoid levels were measured although the adrenal cortex secretes androgen as well as glucocorticoid upon ACTH stimulation [4]. In this study, we showed that CIRCI is associated with significantly lower DHEAS/cortisol ratio and significantly higher inflammatory cytokines and hospital mortality (Table 4). Additionally, the DHEAS/cortisol ratio had excellent capacity to predict hospital survival in cirrhotic patients with septic shock (AUROC = 0.925), suggesting that these two tightly coordinated hormones should be assessed in concert to better reflect adrenal dysfunction. Although administration of low doses of glucocorticoid in septic patients can restore vascular hyporeactivity [46] and reverse the shock status [26, 47–50], its impact on survival remains controversial [26, 48–50]. The reasons for the discrepancy are unclear. Indeed, there are more episodes of superinfection, and higher rates of severe hyperglycemia associated with glucocorticoid administration [26, 49, 50]. In this regard, DHEA has been shown to enhance immunity and help overcome the catabolic effects of glucocorticoid [29]. It is plausible to speculate that glucocorticoid treatment alone may be incomplete unless the imbalance of steroidogenesis is offset by adrenal androgen in addition to glucocorticoid. Further studies are needed to clarify this issue.

Finally, another finding deserving discussion is that cirrhotic patients with septic shock had significantly lower baseline DHEAS, lower baseline DHEAS/cortisol ratio, and decreased cortisol and DHEAS increments upon ACTH challenge, when compared to the noncirrhotic group. However, the baseline cortisol levels were comparable between both groups. This phenomenon can be interpreted as a shift of steroidogenesis toward glucocorticoid biosynthesis at the expense of adrenal androgen in liver cirrhosis. We speculate that liver cirrhosis per se represents a risk factor for altered adrenal steroidogenesis, which may portend a poor prognosis for septic shock if upregulation of glucocorticoid becomes unopposed by adrenal androgen but is still insufficient to control inflammation. In this regard, glucocorticoid resistance may contribute to adrenal dysfunction in our patients. Finally, hepatoadrenal syndrome [27, 28, 51] may need to be redefined and addressed in the context of coordination between separate adrenal hormones.

There are limitations in our study. First, the number of patients is small. Second, the association does not indicate causality because of the observational design. Further studies with a larger cohort and therapeutic intervention are needed in the future.

## Conclusions

We demonstrated a divergent biosynthesis between cortisol and DHEAS in cirrhotic patients with septic shock. This phenomenon, as evidenced by an altered DHEAS/cortisol ratio, is associated with more severe diseases and higher levels of inflammatory cytokines and can serve as a prognostic marker. More investigations are needed to evaluate the role of adrenal androgen in this clinical setting.

## Abbreviations

CIRCI: Critical illness-related corticosteroid insufficiency
DHEAS: Dehydroepiandrosterone sulfate
HPA: Hypothalamic–pituitary–adrenal
ICU: Intensive care unit
MELD: Model for End-Stage Liver Disease
SOFA: Sequential Organ Failure Assessment
SST: Short corticotropin stimulation test
